# Bioengineering strategies for off-the-shelf, cell-based cancer immunotherapy

**DOI:** 10.1007/s44258-026-00099-1

**Published:** 2026-11-05

**Authors:** Brendan R. Barlow, Ana M. Sandoval-Castellanos, Robert J. Canter, Jinhwan Kim

**Affiliations:** 1 Department of Biomedical Engineering, University of California Davis, Davis, CA 95616 USA; 2 Department of Surgery, School of Medicine, University of California Davis, Sacramento, CA 95817 USA; 3 Center for Bioengineering in Medicine, School of Medicine, University of California Davis, Sacramento, CA 95817 USA; 4 Department of Ophthalmology and Vision Science, School of Medicine, University of California Davis, Sacramento, CA 95817 USA

**Keywords:** Off-the-shelf immunotherapy, Cell therapy, In vivo cell engineering, Bioengineering, Cancer immunotherapy

## Abstract

Cancer immunotherapy has reshaped modern oncology by leveraging immune effector cells to recognize and eliminate malignant tissues. Among cell-based approaches, adoptive cell therapies, most prominently chimeric antigen receptor (CAR) T cells, have produced high response rates and deep remissions in several hematologic malignancies. Despite these clinical successes, broad deployment remains limited by the dominant autologous manufacturing paradigm, which is associated with high per patient cost, prolonged vein to vein time, and heterogeneity in starting material and final product quality, particularly in heavily pretreated patients. Off-the-shelf strategies aim to improve scalability and access by standardizing either the cellular input, such as donor derived or renewable sources, or the engineering reagent used to program immune cells. Here, we review bioengineering approaches enabling three complementary off-the-shelf platforms, including allogeneic CAR T cells, natural killer and invariant natural killer T cell-based therapies, and in vivo CAR generation. Lastly, we outline the clinical translation outlook for off-the-shelf cell therapy, highlighting current limitations and future bioengineering and manufacturing directions. Together, these approaches highlight convergent design principles that will shape the next generation of broadly accessible cell-based cancer immunotherapies, including immune compatibility, controllable activation, and manufacturability.

**Graphical Abstract:**

**Figure:**
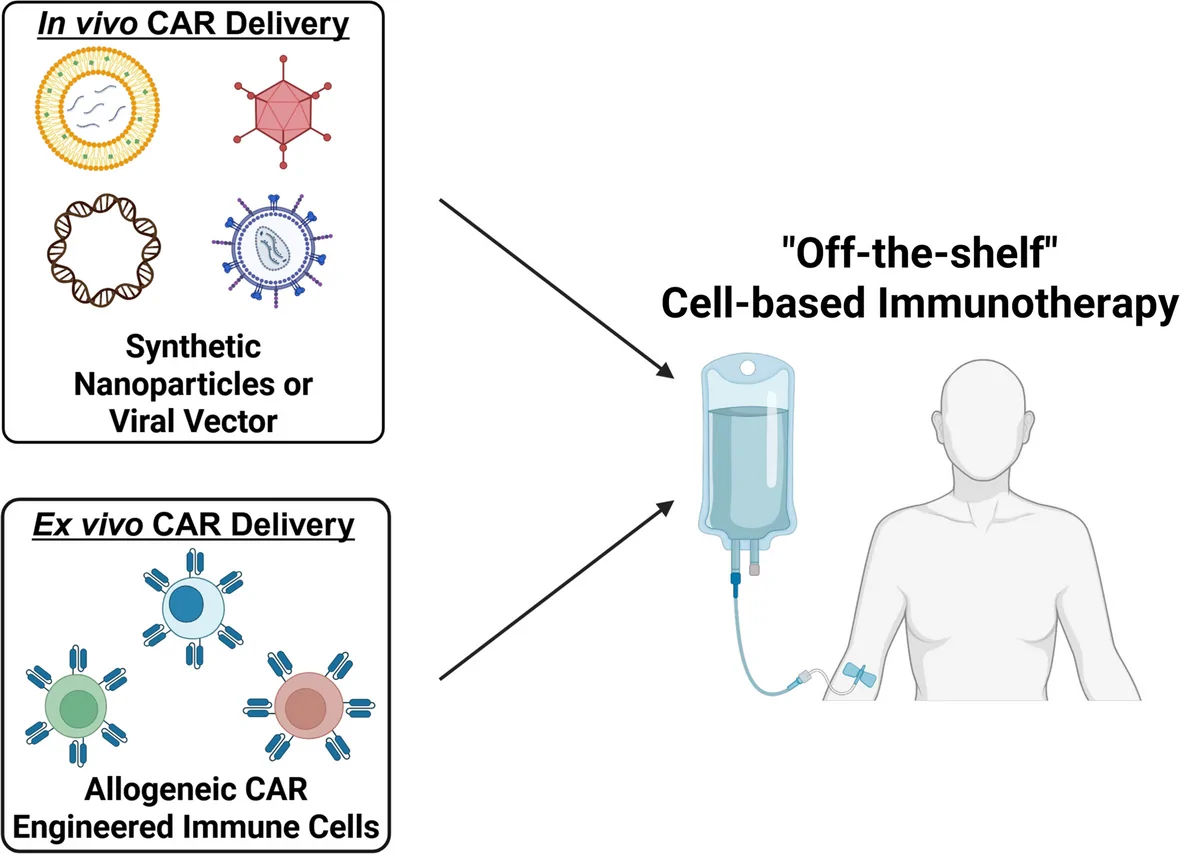

## Highlights

- Autologous CAR T therapy offers strong results but faces various challenges, driving off-the-shelf alternatives.
- This review details three off-the-shelf CAR platforms: allogeneic T cells, NK/iNKT cells, and in vivo CAR generation.
- Off-the-shelf cell therapy translation requires balancing safety, durability with scalable, consistent manufacturing.

## Introduction

Cell-based cancer immunotherapies have transformed the clinical oncology treatment landscape. By using the immune system’s natural ability to adapt and target specific threats, these treatments offer a way to control tumors long-term. Adoptive cell therapies have demonstrated unprecedented clinical efficacy in hematologic malignancies, achieving deep and sometimes long-lasting remissions in patients with otherwise unmanageable disease. Most notably, chimeric antigen receptor (CAR) T cell therapy has drawn significant attention since the first CAR T cell therapy was approved by the U.S. Food and Drug Administration (FDA) in 2017 for treatment of pediatric and young adult patients with acute lymphoblastic leukemia [1]. Since then, several additional CAR T cell therapies have received FDA approval for other hematologic malignancies including B cell leukemia, non-Hodgkin lymphoma, and multiple myeloma [2–8].

In canonical antigen recognition, T cells detect tumor-associated antigens presented by the major histocompatibility complex (MHC) via the T cell receptor (TCR), allowing them to destroy tumorigenic cells selectively. However, tumor cells frequently downregulate MHC-I expression to avoid recognition by the immune system [9–15]. CARs were developed to bypass this escape mechanism by implementing engineered synthetic cell surface receptors that bind to a selected surface antigen in target tumor cells, generating a powerful immune response independent of MHC presentation [2, 3, 16]. These synthetic receptors couple antigen binding to potent intracellular activation signaling, thereby redirecting cytotoxicity toward target tumor cells. This concept has demonstrated the highly potent and even curative potential of cell-based cancer immunotherapy, representing a paradigm shift in the oncology field compared to the simple regression of tumor growth achieved with conventional cancer therapeutics. Despite these successes, the dominant autologous ex vivo manufacturing paradigm limits scalability and access, motivating the development of “off-the-shelf” products that can be manufactured in advance, standardized, and administered on demand.

Current clinical CAR T therapies are predominantly autologous, which reduces the risk of graft-versus-host disease (GvHD). However, the autologous, ex vivo CAR T cell manufacturing procedure is a personalized, multi-step process that can take considerable time. Generally, it begins with the isolation of peripheral blood mononuclear cells (PBMCs) from the patient through leukapheresis. After this, T cells are then isolated from the PBMCs, and specific T cell subsets are chosen [17–19]. The selected T cells are then activated to stimulate their proliferation and genetically modified to express the CAR construct. The CAR T cells are then expanded for 4 to 14 days to reach sufficiently high numbers for treatment [17, 18, 20]. The resulting product then must undergo rigorous quality control testing before it can be clinically used. Upon passing these criteria, the CAR T cell product is either used fresh or cryopreserved and stored until the patient is ready for infusion.

This complex manufacturing workflow required for each individual patient imposes substantial limitations that restrict the broader clinical impact of these therapies. First, it is extremely costly, typically costing over $400,000 per patient, which poses extreme financial burden on patients and restricts access [21]. Second, the time from collection of a patient’s white blood cells (leukapheresis) to infusion with engineered CAR T cells, known as the vein-to-vein time, can be upwards of 40 days due to the manufacturing time, quality control tests, and other logistical challenges [22, 23]. During this time, patients’ disease will continue to progress, which can lead to lower complete response rates to the treatment [24]. Third, the individualized nature of CAR T cell production leads to significant heterogeneity in the final product, which contributes to the wide-ranging patient outcomes of CAR T therapy. Patients eligible for CAR T therapy are often heavily pretreated with chemotherapy, radiation, or other immunosuppressive reagents, which are known to reduce absolute T cell counts and impair T cell function [3, 25, 26]. Consequently, insufficient expansion of the T cells and failure of the cells to pass quality control tests can occur, ultimately resulting in a relatively high manufacturing failure rate up to 10% [3, 22, 27].

Beyond manufacturing failures, substantial inter-patient variability is observed in the clinical performance of autologous CAR T cell products. Several studies have shown that the phenotype and functional quality of the patient-derived T cell population strongly influence CAR T expansion, persistence, and therapeutic efficacy. In one study by Fraietta et al., the authors noted that patients with chronic lymphocytic leukemia who received CAR T cell therapy and achieved complete remission possessed CAR T cells enriched with memory-related gene expression, while non-responder’s CAR T cells had upregulation of exhaustion markers [28]. Other studies have also noted various phenotypic markers of CAR T cells that seem to correspond with favorable or unfavorable treatment outcomes [29–31], suggesting that the patient’s intrinsic immune biology plays an important role in autologous CAR T therapy success. However, these phenotypes are not solely patient-driven, as CAR construct design can also shape T cell phenotype [32], and prolonged ex vivo culture during manufacturing can promote T cell exhaustion [33]. Therefore, autologous CAR T treatment outcomes likely reflect a combination of patient biology, construct design, and manufacturing conditions, complicating efforts to produce an optimal and replicable therapeutic.

**Fig. 1:**
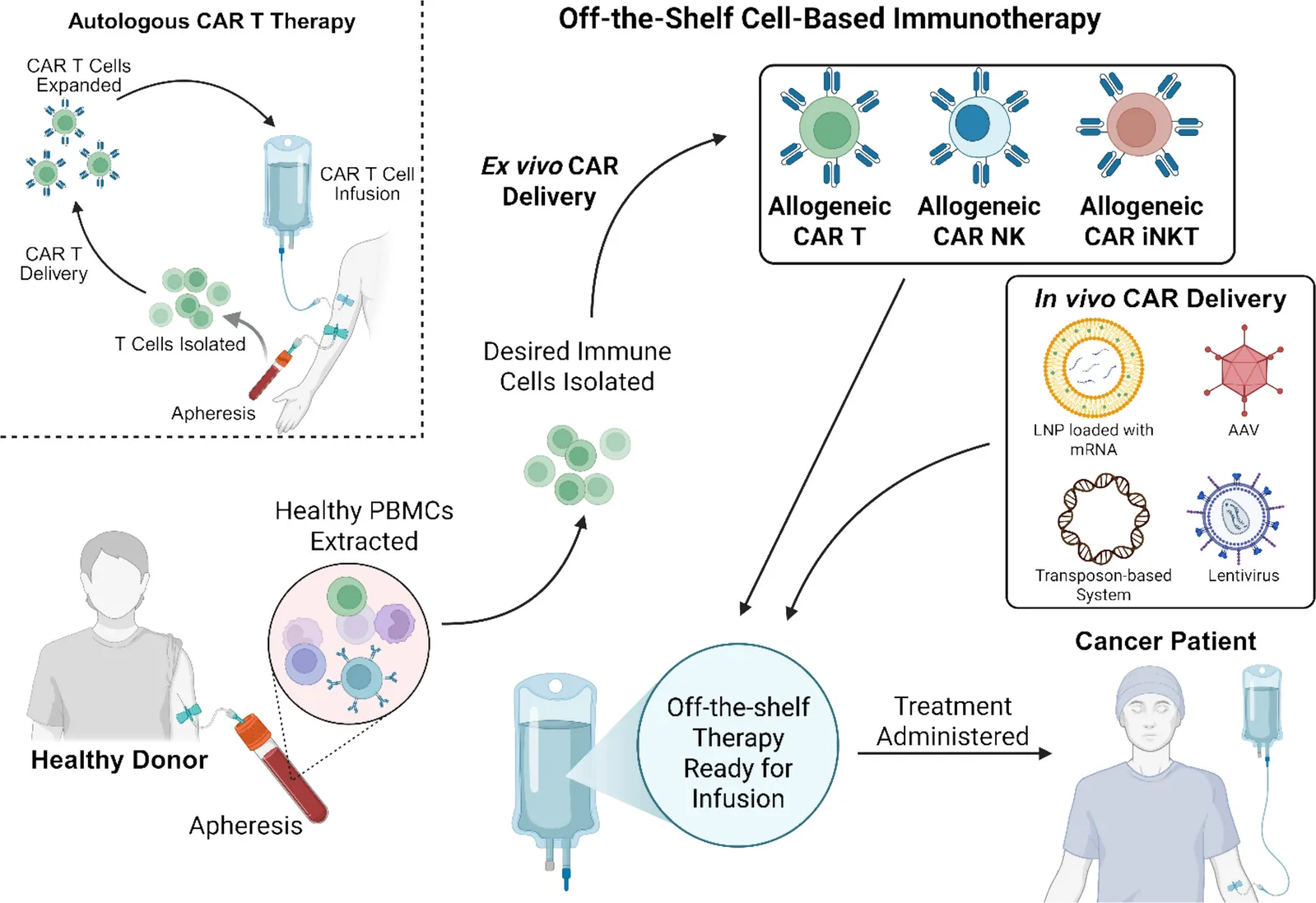
Overview of autologous versus off-the-shelf cell-based immunotherapy approaches for cancer treatment. Autologous chimeric antigen receptor (CAR) T therapy (top left) uses cells isolated from the patient themselves that are engineered ex vivo to express a CAR, expanded, and reinfused into the same patient. In contrast, off-the-shelf strategies use either healthy donor cells enhanced ex vivo to improve their tumor targeting capabilities, or in vivo delivery approaches, where the patient’s immune cells are engineered directly within the patient. Both off-the-shelf approaches provide scalable and rapidly accessible alternatives to personalized autologous therapies. Abbreviations: AAV, adeno-associated virus; iNKT, invariant natural killer T; LNP, lipid nanoparticle; NK, natural killer; PBMCs, peripheral blood mononuclear cells. Created in BioRender. Barlow, B. (2026) https://BioRender.com/kszzzad

These challenges with autologous T cell therapy have led to an emerging demand for “off-the-shelf” cell-based immunotherapy platforms that are ready-to-use, scalable, and broadly accessible. In principle, off-the-shelf products can be manufactured in large batches from healthy donor cells, or in some cases, from standardized renewable sources like induced pluripotent stem cells, reducing the production time and cost, and improving the product consistency relative to current autologous approaches. To compare off-the-shelf strategies without assuming a single best solution, it is useful to view the field through three recurring design bottlenecks that different platforms address with distinct tradeoffs. First, donor-derived T cell products must manage alloreactivity and immune rejection, which can limit persistence through immune clearance [27]. Second, effector-cell intrinsic biology shapes safety and function: natural killer (NK) cells offer an inherently low GvHD risk after allogeneic transfer, but often require engineering to improve persistence, trafficking, and activation in vivo, particularly in solid tumors [34]; invariant natural killer T (iNKT) cells share this low GvHD risk while also providing innate-like antitumor activity and the capacity to reprogram the immunosuppressive tumor microenvironment (TME), though their low frequency in PBMCs makes therapeutic expansion challenging [35]. Third, manufacturing and logistics influence scalability and time-to-treatment across ex vivo-engineered products, motivating interest in in vivo CAR generation as an alternative delivery paradigm that shifts some complexity from cell manufacturing to targeted gene delivery and expression control. Here, we focus on these three leading off-the-shelf platforms: 1) allogeneic CAR T cells, 2) NK and iNKT cell-based therapies, and 3) in vivo CAR delivery, as complementary approaches that prioritize these bottlenecks differently, highlighting bioengineering strategies to improve safety, persistence, and specificity, and concluding with an outlook on how these strategies may converge to redefine the next generation of cell-based cancer immunotherapies (Fig. 1).

## Bioengineering strategies for allogeneic CAR T cell therapy

Allogeneic CAR T cell therapies have recently emerged as one strategy to overcome the limitations arising from autologous cell-based therapy. By using healthy donor-derived immune cells expanded ex vivo, allogeneic approaches enable the generation of standardized, readily available, off-the-shelf CAR T products. This could streamline the manufacturing procedure, therefore reducing cost and improving accessibility, while also reducing the waiting period for patients that require treatment. However, the use of non-self T cells introduces the risk of GvHD, TCR expressed on the surface of the infused T cell recognizes the mismatched host human leukocyte antigen (HLA) class I molecules, leading to the infused T cells activating against healthy host tissues and causing tissue damage [36]. In addition to GvHD, infused T cells are susceptible to clearance by the host, as the host T cells can recognize the foreign HLA molecules on the donor T cells and eliminate them. This host-versus-graft (HvG) reaction causes allogeneic CAR T cell therapy to have significantly lower persistence compared to autologous methods. Therefore, the central bioengineering challenge in allogeneic CAR T is to decouple CAR-mediated tumor recognition from endogenous alloreactivity while maintaining in vivo persistence. Most reported approaches converge on two design philosophies: 1) gene engineering/editing to ablate TCR and/or HLA-related pathways, and 2) non-genetic modulation of receptor signaling or antigen presentation to reduce alloreactivity with fewer permanent edits. The following sections describe these strategies and the trade-offs that shape their translational potential.

### Gene editing approaches

Numerous gene editing strategies have been developed to create allogeneic CAR T cells with lower risk of GvHD and HvG reactions. A major first step that many allogeneic CAR T cell studies have employed is a knockout of the TCR to prevent targeting of healthy tissues by the CAR T cells [37–45]. In an early attempt to make allogeneic CAR T cells, Torikai et al. used a gene editing strategy involving zinc finger nucleases to remove the TCR α and β chain constant regions, which prevented the T cells from using their TCR to recognize host antigens [39]. By adding a CAR that targets CD19, this study was able to make CAR T cells that could effectively kill malignant B cells while avoiding GvHD. Subsequent studies have expanded upon this approach, such as a study by Eyquem et al., who used gene editing strategies to disrupt TCR α chain (TRAC) expression by inserting a CD19-specific CAR transgene into the TRAC locus. CAR knock-in at the TRAC locus allows CAR expression to be controlled by endogenous TCR regulatory elements, resulting in more uniform CAR expression and higher CAR T cell potency [45, 46]. TCR knockout in CAR T cells has been applied in human clinical trials for treatment of B cell malignancies, with a majority of patients responding without GvHD [37, 47, 48].

While TCR disruption can prevent GvHD, allogeneic CAR T cells remain vulnerable to elimination by the host immune system [3, 49]. Host T cells can recognize a mismatch in the donor T cells HLA class I molecules, triggering them to kill the infused donor T cells [3, 50]. To deal with this limitation, researchers frequently knockout β2-microglobulin (B2M) expression [3, 40, 49–54]. The B2M protein is a piece of the HLA class I molecule, and by inactivating the gene encoding it in donor T cells, the HLA class I surface protein cannot be expressed, allowing the donor cell to evade host T cells [40]. Many attempts at creating allogeneic CAR T cells have since begun eliminating this protein as part of the process towards creating universal CAR T cells. For instance, in a study by Ren et al., CRISPR/Cas9 was used to disrupt expression of B2M, along with TCR and PD-1 expression, in allogeneic CAR T cells [53]. These disruptions were used to create less alloreactive, more effective allogeneic CAR T cells. The authors found that knocking out B2M was sufficient to generate HLA-I negative CAR T cells, and that, combined with the other gene modifications made, these cells had significantly reduced alloreactivity in vivo [53]. Additionally, the gene-disrupted CAR T cells maintained their antitumor efficacy compared to non-gene disrupted CAR T cells. This study, along with many others demonstrating the effect of B2M knockouts in CAR T cells, supports that B2M knockout could be a step towards creating immune-evading allogeneic CAR T cells.

**Fig. 2:**
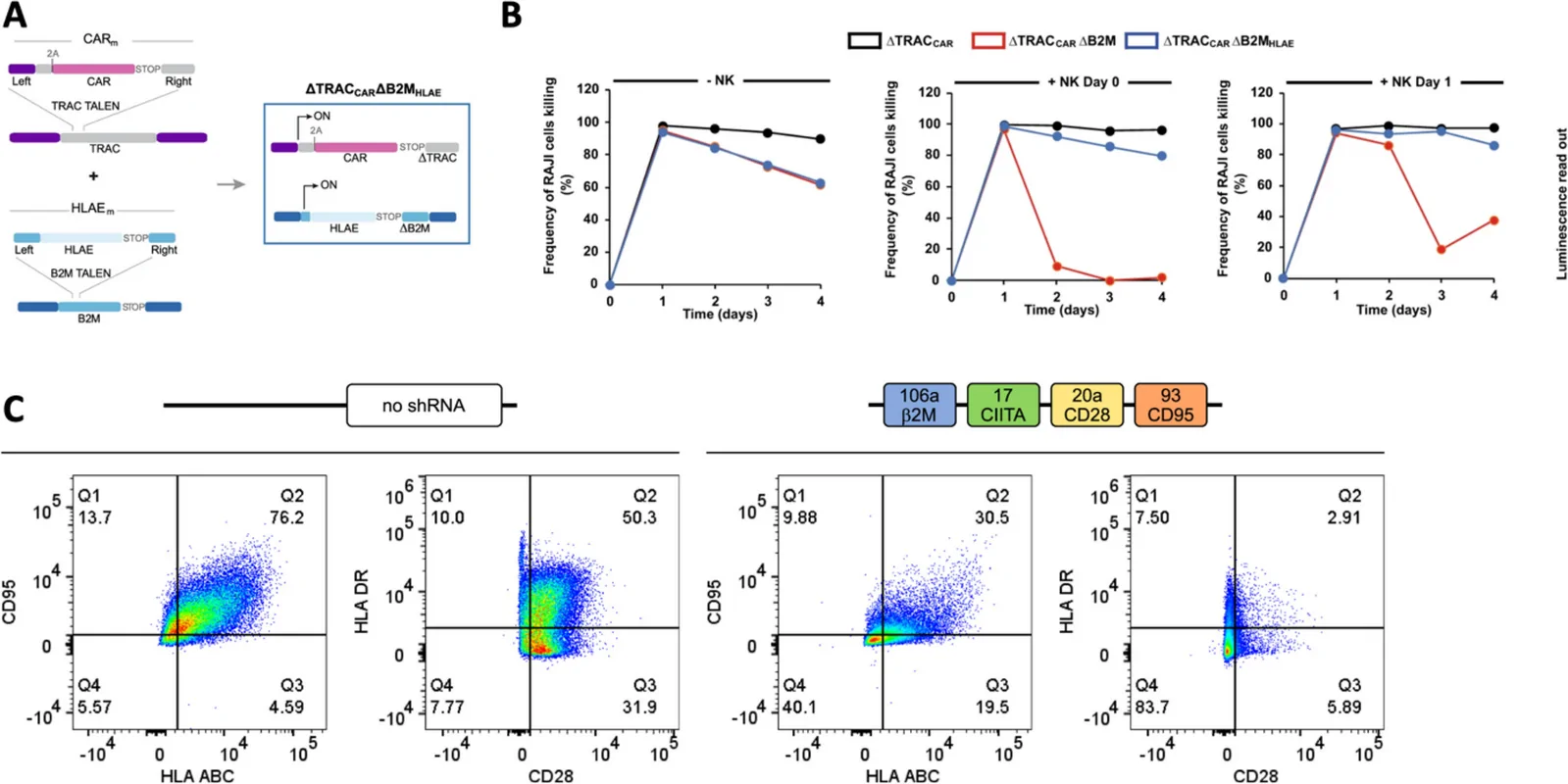
Gene editing and non-gene editing approaches to create allogeneic CAR T cell therapy. **A** Schematic showing gene insertions used in Jo et al. study to disrupt T cell receptor (TCR) and Human leukocyte antigen (HLA) class I expression while introducing CAR and NK-inhibitory receptor HLA-E expression [40]. **B** Graphs of genetically modified CAR T cell killing activity on target leukemia cell line (RAJI). In each scenario, CAR T cells were at a ratio of 2.5:1 CAR T:RAJI cells. The graphs show killing effects in the presence or absence of NK cells at NK to CAR T cell ratio of 1:1, which were added either on Day 0 or Day 1 [40]. Reproduced with permission [40]. Copyright 2022, Springer Nature. **C** Flow cytometry assessing expression of genes targeted for RNA interference to generate less alloreactive CAR T cells. Delivery of the multiplex short hairpin RNA (shRNA) platform significantly reduced expression of all target genes compared to no shRNA control, creating less alloreactive CAR T cells [56]. Reproduced with permission [56]. Copyright 2023, Elsevier Inc

However, while knockout of B2M can reduce donor CAR T recognition by host T cells, it also exposes them to NK cell-mediated “missing self” recognition and clearance. To prevent recognition by NK cells, some studies have combined previously mentioned gene editing approaches with the addition of the inhibitory NK receptors HLA-E or HLA-G [40, 52, 54]. HLA-E is known to prevent NK cell response when expressed in pluripotent stem cells with missing HLA class I molecules by binding to NKG2A receptors, which are expressed on most NK cells [51, 55]. This approach was implemented in a study by Jo et al., which used gene editing to disrupt the TRAC and B2M loci in T cells while inserting transgenes for a CAR and HLA-E (Fig. 2A) [40]. This gene modification created CAR T cells which were TCR and HLA class I deficient while being capable of avoiding NK cell missing-self recognition. In vitro, this modification allowed the cells to evade activated NK cells and maintain their antitumor efficacy (Fig. 2B). The researchers also tested the modified cells in vivo using immunodeficient mice engrafted with human PBMCs containing NK cells [40]. These engineered cells showed significantly reduced NK cell activation and clearance compared to HLA-E negative CAR T cells, supporting the potential feasibility of this strategy to generate off-the-shelf CAR T therapies.

### Non-gene editing approaches

While gene editing approaches represent a major focus for generating allogeneic CAR T cells, several non-gene editing engineering approaches are also being explored to create CAR T cells with low alloreactivity. These approaches reduce the complexity introduced with editing multiple genes, and focus on inhibiting the expression of alloreactive receptors instead of completely eliminating them [27]. For example, in a study by Michaux et al., the authors used a non-gene editing approach to disrupt the TCR component CD3ζ, which prevented infused allogeneic CAR T cells from triggering GvHD [57]. To do this, they transduced donor T cells with a retroviral vector coding for a truncated CD3ζ peptide termed a T cell inhibitory molecule that incorporates into the TCR complex and blocks TCR signal from activating the T cell, while allowing activation by the transduced CAR component. These T cells still expressed the TCR on their surface, but did not cause GvHD as the TCR signal was impaired [57]. Additionally, the TCR-inhibited CAR T cells still generated powerful antitumor activity against the target cells in vitro and in vivo. This study demonstrates one example of how non-gene editing methods could be used to make safe and effective allogeneic CAR T cells while reducing the complexity involved in manufacturing them.

Another non-gene editing method demonstrated previously to generate safe allogeneic CAR T cells is the use of RNA interference with small non-coding RNAs to disrupt TCR [56, 58]. For instance, Rossi et al. used a short hairpin RNA (shRNA)-based multiplex system to silence CD3ζ and B2M, as well as CD28, CD95, and CIITA (Fig. 2C) [56]. Silencing CD3ζ and B2M, as mentioned previously, reduces GvH and HvG reactions. CD28 knockdown further dampens TCR signaling and limits excessive T cell activation in their particular CAR T cell construct, while CD95 silencing reduces host immune targeting of the donor CAR T cells, increasing persistence [56]. Finally, CIITA controls expression of HLA class II genes, so by knocking down CIITA, HLA class II expression is reduced [59]. This further improves immune evasion and increases persistence in the host. Together, this multiplex RNA interference strategy enables precise control of T cell activation without requiring direct genome modification, supporting the development of scalable and safer CAR T cell therapies.

Several of the allogeneic CAR T cell generation strategies discussed above have now advanced into clinical trials. Early-phase trials of UCART19, a TRAC/CD52-disrupted allogeneic CD19-targeting CAR T product, enrolled both pediatric and adult patients with relapsed/refractory acute lymphoblastic leukemia, providing some of the first clinical evidence that allogeneic CAR T cells could be administered without GvHD (NCT02746952, NCT02808442) [37, 47, 60]. The ALPHA/ALPHA2 clinical trials also generated and used CD19-targeted allogeneic CAR T cells for the treatment of relapsed/refractory large B-cell lymphoma (NCT03939026, NCT04416984) [61]. The reported overall and complete response rates for these trials were 58% and 42%, respectively, with a median duration of complete response of 23.1 months and no cases of GvHD, immune effector cell-associated neurotoxicity syndrome, or grade 3 or higher cytokine release syndrome (CRS) [61]. In the Phase I UNIVERSAL clinical trial, an anti-B-cell maturation antigen (BCMA) allogeneic CAR T therapy called ALLO-715 was evaluated for multiple myeloma and demonstrated a 56% overall response rate with manageable toxicity and no GvHD (NCT04093596) [62]. The median time to receive allogeneic CAR T cells after enrollment was only 5 days, representing a significant shift in treatment time compared with typical autologous CAR T timelines. While these results are encouraging, they reflect early-phase conditions, and durability of response and long-term persistence of allogeneic CAR T cells remain active areas of investigation as the field moves toward later-stage trials.

Across reported allogeneic CAR T cell strategies both preclinically and clinically, a consistent trade-off emerges between manufacturing simplicity, immune compatibility, and in vivo persistence. A minimal edit set, such as TCR disruption alone, can substantially reduce GvHD risk while simplifying manufacturing, but infused cells often remain vulnerable to HvG immune clearance, limiting persistence and durability [27, 36]. Adding HLA class I ablation such as B2M knockout can reduce T cell–mediated rejection and improve persistence [3, 40, 49–54], but it can also increase susceptibility to NK cell “missing-self” recognition and clearance. To rebalance this, some approaches introduce inhibitory ligands such as HLA-E or HLA-G, which can mitigate NK-mediated elimination [40, 52, 54], but add additional engineering steps and product complexity that may affect scalability and regulatory burden. In parallel, non-gene editing approaches (e.g., dominant-negative signaling components or RNA interference) can reduce permanent genomic modifications and streamline manufacturing; however, incomplete or transient suppression of alloreactive pathways may still permit residual alloreactivity or immune recognition, particularly in settings requiring prolonged persistence. Overall, these constraints help explain why durable, universally compatible allogeneic CAR T products remain challenging and motivate the exploration of effector cell types that reduce alloreactivity through cell-intrinsic biology rather than multi-gene engineering.

## Bioengineering of allogeneic NK cell therapies

Another method of creating off-the-shelf immunotherapies exploits NK cells’ natural ability to target cancer cells. NK cells are cytotoxic component of the innate immune system, with capacity to destroy malignant cells and recruit other immune cells without prior antigen sensitization and has significantly lower risk of CRS over T cell-based therapies [63]. From an off-the-shelf perspective, NK cells are particularly attractive because they do not rely on antigen-specific TCR recognition for activation, and therefore have a substantially reduced baseline risk of GvHD after allogeneic transfer compared with donor T cell products. At the same time, NK cell therapies often face distinct biological bottlenecks: most notably limited persistence, suboptimal trafficking and tumor infiltration, and functional suppression within the TME. All of these bottlenecks motivate bioengineering strategies aimed at enhancing durability and tumor engagement while preserving favorable safety.

NK cells are known to naturally exhibit antitumor activity, and thus several early clinical trials attempted to use autologous NK cells expanded ex vivo to treat both liquid and solid cancers [64–69]. These trials found that autologous NK cell infusion is safe, but the adoptively transferred cells exhibited limited antitumor activity due to self-MHC recognition on cancer cells by the NK cells [64]. Additionally, autologous NK cell therapy suffers from some of the same challenges as autologous T cells, as prior chemotherapy treatments deplete NK cell counts as well as impairing their function [34, 70]. Due to these constraints, there was a shift towards using allogeneic NK cell transfer instead, where cells can be sourced from healthy donors and manufactured in advance. However, reduced alloreactivity alone does not guarantee durable clinical efficacy. Particularly in solid tumors settings, adoptively transferred NK cells can exhibit limited persistence, inadequate trafficking into tumor sites, and diminished function under immunosuppressive conditions (e.g., inhibitory ligands, soluble suppressive factors, metabolic stress, and hypoxia in the TME) [71]. As a result, the major bioengineering objective for NK platforms is not primarily to “turn off” alloreactivity (as in allogeneic CAR T), but rather to increase specificity and functional activity while improving durability in vivo.

One widely explored strategy to address these constraints is to engineer NK cells with CARs, enabling antigen-directed recognition and cytotoxicity. Since adoptively transferred allogeneic NK cells have a low risk of causing GvHD, CAR-engineered NK (CAR NK) cells could provide a potentially safer off-the-shelf alternative compared with allogeneic CAR T cells, while still enabling programmable specificity [72]. Additionally, CAR NK cells retain native CAR-independent tumor surveillance mechanisms (e.g., activation through endogenous NK receptors), providing redundancy in tumor recognition beyond a single engineered target antigen [73, 74]. While CAR T cells have been the primary focus of CAR research, multiple studies have demonstrated the potent anti-tumor efficacy of CAR NKs in a variety of tumor types, supporting CAR NK as a credible off-the-shelf platform (Table 1) [72, 75–91]. For instance, for the treatment of B cell malignancies, Marin et al. designed an anti-CD19 CAR NK cell to eliminate CD19^+^ B cells and tested it in a human clinical trial [75]. The researchers noted no significant toxicities such as GvHD or CRS, and treatment response rates comparable to CAR T cell therapy responses. This clinical experience is noteworthy because it supports a core promise of the NK platform: antigen-specific targeting can be introduced while maintaining a favorable safety profile in an allogeneic setting.

**Table 1:** Studies using CAR NK cells

CAR Target	Cancer Type	CAR Delivery Method	Study and Notes
CD19	B-cell lymphoma	Retroviral vector	Clinical trial with 37 participants. CAR NKs Demonstrated efficacy similar to autologous anti-CD19 T cells but with improved safety profile [75]
			[76]
		mRNA transfection	NK cells were transfected with mRNA encoding an anti-CD19 CAR using electroporation [77]
		iPSC differentiation platform	[78]
CD20	B-cell malignancies	Retroviral vector	Anti-CD20 CAR NK cells effectively lysed otherwise NK-resistant primary leukemias and lymphomas [79]
CD33	Acute myeloid leukemia	Lentiviral vector	[80]
			[81]
HER2	Glioblastoma	Lentiviral vector	[72]
	Breast carcinoma, Ovarian carcinoma, Melanoma, Renal cell carcinoma	Lentiviral vector	Transduced NK cells exhibited significant antitumor activity against various HER2 (ErbB2) expressing tumors in vitro and in vivo [82]
	Breast carcinoma, Ovarian carcinoma, Squamous cell carcinoma	Retroviral vector	[83]
GD2	Neuroblastoma	Retroviral vector	[84]
	Glioma	Lentiviral vector	[85]
	Melanoma	CRISPR-Cas9	Used non-viral gene editing technique to insert anti-GD2 CAR, which demonstrated cytotoxicity to melanoma cell line in vitro [86]
Robo1	Pancreatic carcinoma	Lentiviral vector	[87]
	NSCLC		[88]
	NSCLC, Esophageal squamous cell carcinoma, Colon cancer		[89]
ROR1	Neuroblastoma	mRNA transfection	Anti-ROR1 CAR NK cells demonstrated enhanced antitumor activity against neuroblastoma cell lines in the presence of N803, an IL-15 superagonist drug [90]
MCAM	Neuroblastoma	mRNA transfection	[91]

CAR NK approaches have also been evaluated in solid tumors, where safety and delivery route may be particularly important due to anatomic constraints and the immunosuppressive TME. Burger et al. described CAR NK cells engineered to target glioblastoma [72]. In this study, NK cells were transduced with a CAR targeting the protein HER2, which is known to be overexpressed in glioblastoma [92, 93]. These CAR NK cells were then used to treat nine patients with recurring glioblastoma by intracranial injection near the patients’ tumor at various doses. All patients experienced no severe side effects, and overall survival was much longer in two of the three patients that received the highest dose of CAR NK cells (1 × 10^8^ cells) compared to the rest of the cohort [72]. In another example of using CAR NK against solid tumors, a study by Chu et al. demonstrated successful production of anti-ROR1 (receptor tyrosine kinase-like orphan receptor 1) CAR NK cells for treatment of neuroblastoma. These CAR NKs demonstrated effectiveness for tumor growth suppression and long-term survival in a neuroblastoma xenograft mouse model [90]. These studies, along with numerous others demonstrating the performance of CAR NKs, verify that CAR NK therapy can be effective, with an improved safety profile compared to T cell therapies due to the reduced risk of GvHD and CRS.

**Fig. 3:**
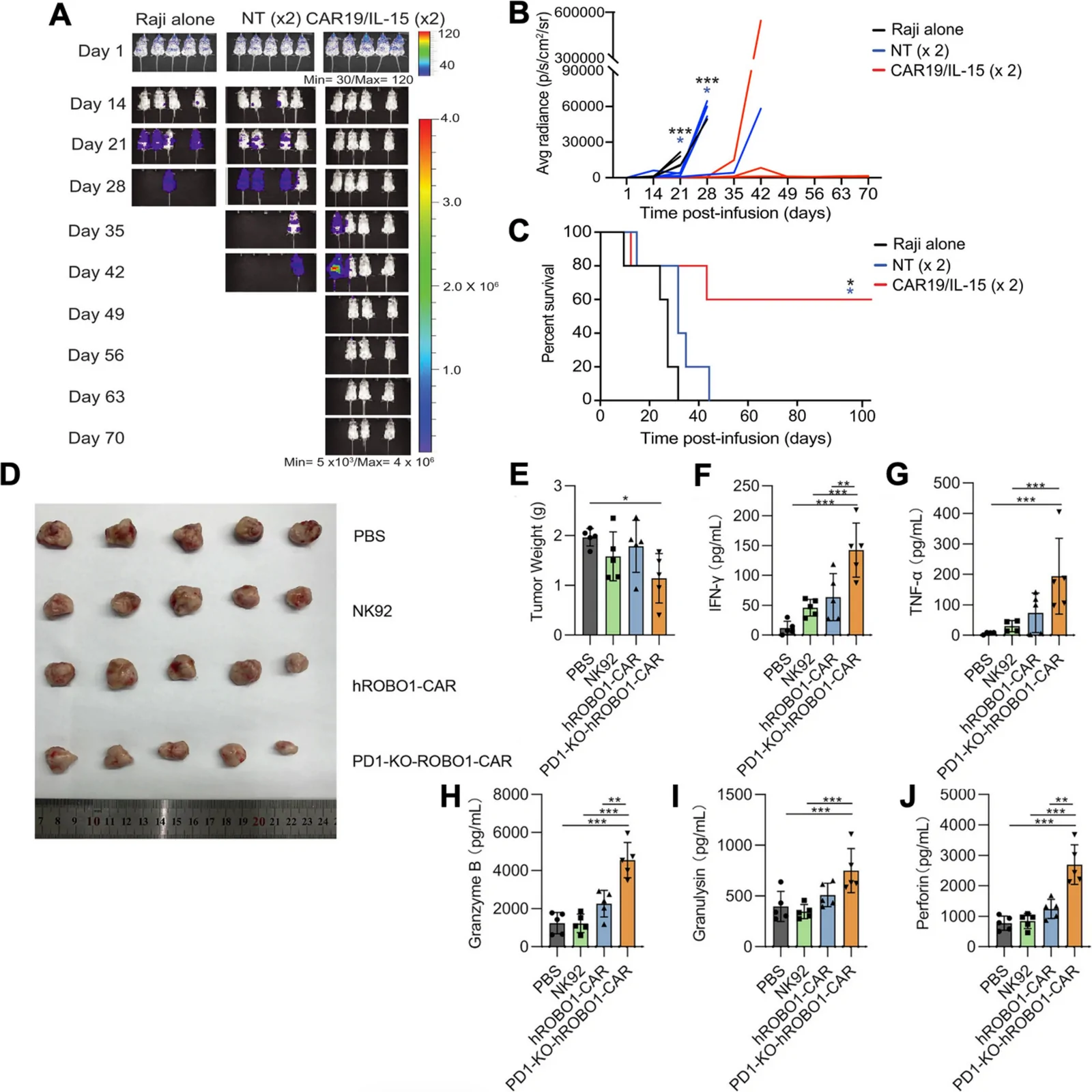
In vivo antitumor efficacy of CAR NK cells in preclinical cancer models. **A**-**C** IL-15 expressing anti-CD19 CAR NK cells (CAR19/IL-15) demonstrated superior tumor control in an NSG Raji lymphoma xenograft model compared to non-transduced (NT) NK cells and untreated controls. **A** Bioluminescence imaging showing tumor progression over 70 days of luciferase-labeled Raji lymphoma xenograft across treatment groups. **B** Quantification of average radiance (p/s/cm^2^/sr) over time from bioluminescence imaging (*n*=5, mice per groud) demonstrating significantly reduced tumor burden in CAR19/IL-15-treated mice compared to Raji alone and NT control groups. **C** Kaplan–Meier survival curves showing prolonged survival in CAR19/IL-15-treated mice compared to NT and Raji-alone groups. Reproduced with permission [76]. Copyright 2023, American Association for the Advancement of Science.*P ≤ 0.05 and **P ≤ 0.01. Black asterisks denote Raji alone versus CAR19/IL-15; blue asterisks denote NT versus CAR19/IL-15. *P* values for average radiance (B) were determined by unpaired t test, and P values for survival (C) were determined by log-rank (Mantel-Cox) test. **D**-**J** ROBO1-targeted PD-1 knockout CAR NK cells (PD1-KO-hROBO1-CAR) demonstrated superior antitumor efficacy in a subcutaneous non-small cell lung cancer (NSCLC) xenograft model. **D** Representative image of excised tumors from NSG mice bearing H1299 subcutaneous xenografts following treatment with PBS (vehicle control), unmodified NK-92, ROBO1-targeting CAR NK cells, or PD1-KO-hROBO1-CAR NK-92. **E** Tumor weights at Day 35, when treatment was ended across treatment groups (*n*=5). **F**-**J** Serum concentrations of markers for immune response and tumor suppression at Day 26 including (**F**) IFN-γ (**G**) TNF-α (**H**) Granzyme B (**I**) Granulysin and (**J**) Perforin (*n*=5). The PD1-KO-hROBO1-CAR NK-92 cells led to significantly improved tumor suppression and immune activation compared to all other groups. *P<0.05, **P<0.01, and ***P<0.001. *P* values were determined with one-way ANOVA with post hoc correction. Reproduced with permission [88]. Copyright 2025, Springer Nature America, Inc

Despite the favorable GvHD profile and often promising results of allogeneic CAR NK cells, HvG rejection, where the patient’s own immune system eliminates the adoptively transferred allogeneic cells, remains a significant barrier to durable in vivo persistence. A strategy implemented in several studies is the introduction of IL-15 expression in the NK cells along with the CAR construct to enhance their proliferation and activation even when the host immune system tries to eliminate them (Fig. 3A-C) [54, 75, 94–97]. In these studies, the IL-15 expressing cells were shown to have increased persistence in the host. Another genetic engineering strategy to avoid host immune detection is the disruption of the B2M gene and introduction of an HLA-E molecule in allogeneic CAR NK cells via CRISPR/Cas9 [98]. Disruption of B2M knocks out surface expression of HLA class I molecules, which makes the allogeneic cells resistant to detection by host T cells. The introduction of HLA-E avoids host NK cell-mediated ‘missing-self’ detection and killing [98]. The persistence of CAR NK cells can also be increased through strategies like knocking out immune checkpoints such as PD1 in the infused NK cells, as done in a study by Tao et al. (Fig. 3D-J) [88]. At the clinical level, lymphodepleting conditioning regimens (e.g., fludarabine/cyclophosphamide) remain the standard approach to transiently suppress host immunity and facilitate engraftment [75, 96]. Integrating these genetic and clinical strategies will likely be necessary to achieve the durable persistence required for robust antitumor responses with off-the-shelf CAR NK products.

The broader clinical landscape for allogeneic CAR NK cells is still in early stages, although the number of trials registered each year has steadily increased [99]. At the moment, most registered trials are Phase I or Phase I/II studies, predominantly in hematologic malignancies such as acute myeloid leukemia, multiple myeloma, and B cell lymphomas [99, 100]. These trials generally report low toxicity from the treatment and efficacy similar to autologous CAR T cell therapy results [75, 96]. There are also an increasing number of clinical trials attempting to use CAR NK cells for the treatment of solid tumors, although results are limited at the moment [101]. In one trial using CAR NK cells for the treatment of colorectal cancer, the treatment was well-tolerated, and there appeared to be a potential synergistic effect between CAR NK cell treatment and anti-PD1 immune checkpoint inhibitor therapy [102]. However, the sample size was small, with only six patients total enrolled, and only three of those receiving anti-PD1 therapy. Therefore, while allogeneic CAR NK therapy for liquid tumors has seen very promising results, further evidence is needed to understand the potential of allogeneic CAR NK therapy for the treatment of solid tumors clinically.

Together, these examples highlight the promise of NK cells as a platform for off-the-shelf cancer immunotherapy. While autologous NK cells are limited by self-MHC recognition and prior treatment-related impairment, allogeneic and bioengineered NK cell approaches have largely overcome these constraints. Genetic modification enables NK cells to not only directly target tumor antigens through CAR expression, but also to reshape the tumor microenvironment by eliminating immunosuppressive cells, enhancing the efficacy of complementary therapies. The improved safety of NK cells compared to T cells due to minimal risk of GvHD and CRS are also highly encouraging. Nevertheless, continued optimization of NK cell engineering and manufacturing is needed to improve durability, trafficking, and resistance to suppressive tumor microenvironments, which are often critical for sustained responses. Limitations in NK cells tumor trafficking and susceptibility to TME-mediated suppression have motivated exploration into other effector cell types that may address these issues without significant engineering, including invariant NKT cells, which will be discussed in the following section.

## Bioengineering of allogeneic CAR iNKT cell therapies

Beyond conventional T and NK cells, iNKT cells have emerged as an alternative potential platform for allogeneic, off-the-shelf cell therapy. NKT cells are a specialized subset of T lymphocytes that express markers characteristic of both conventional T cells, such as a TCR, and NK cells, including NK1.1 [103–105]. However, unlike conventional T cells which recognize peptide antigens with their TCR, NKT cells recognize lipid antigens presented by the MHC-like molecule CD1d [103, 106]. iNKT cells, which are also called type I NKT cells, are the most well-characterized subset of NKT cells, defined by expression of a semi-invariant TCR [103, 107]. iNKT cells are particularly well-suited for allogeneic off-the-shelf therapy, as they innately have a low risk of causing GvHD due to their CD1d-dependent activation [108, 109]. Unlike the highly polymorphic MHC molecules used by conventional T cells that drive alloreactive responses, CD1d is a highly conserved, monomorphic molecule [109, 110]. Since iNKT cells are restricted to antigen recognition using CD1d, allogeneic iNKT cells are unlikely to attack host tissues.

Other innate benefits of iNKT cells are their ability to efficiently traffic to and infiltrate tumors due to their wide repertoire of chemokine receptors [104, 111, 112], and their capacity to reprogram the immunosuppressive TME by targeting CD1d-expressing tumor-associated macrophages and modulating myeloid derived suppressor cells to reduce their immunosuppressive function [108, 113–115]. For instance, one early CAR iNKT study by Heczey et al. demonstrated that iNKT cells expressing a GD2 targeting CAR construct had dual cytotoxicity against neuroblastoma target cells, as well as CD1d-positive tumor-associated macrophages [108]. The benefits and significant potential for an off-the-shelf therapy have led to many preclinical studies seeking to engineer allogeneic CAR iNKT cells for cancer immunotherapy [108, 109, 116–121]. These studies have demonstrated the favorable safety profile and anti-tumor activity of allogeneic CAR iNKT cells against various different cancer types in vitro and in vivo, highlighting their significant potential.

Despite the advantages of iNKT cells as a source for allogeneic CAR therapy, a major practical challenge for developing this therapy is the scarcity of iNKT cells in peripheral blood. The concentration of iNKT cells in peripheral blood is very low, ranging from 0.01% to 1% of all PBMCs, which limits their therapeutic applications [105, 106]. One approach to address this limitation being explored by the Yang lab at University of California, Los Angeles is the use of hematopoietic stem cells to generate large quantities of iNKT cells [117–119, 122]. In a study by Li et al. from the Yang lab, hematopoietic stem cells derived from cord blood were genetically modified with CRISPR-Cas9 to knock out HLA-I and HLA-II, while introducing expression of: an iNKT TCR, a multiple myeloma-targeting CAR construct, and IL-15 [119]. These cells were also found to highly express NK receptors such as NKG2D and DNAM-1. This allowed them to target and kill tumor cells through three mechanisms, using their CAR, the iNKT TCR, and with the NK receptors detecting stress signals, providing them with high anti-tumor efficacy even when the tumor lacks the antigen targeted by the CAR [119]. Additionally, the allogeneic CAR iNKT cells did not cause GvHD, demonstrating their safety in the mouse in vivo model used. In contrast, conventional allogeneic CAR T cells used in the same model caused severe GvHD, leading to death with considerable immune cell infiltration into vital organs [119]. Therefore, while the scarcity of naturally occurring iNKT cells may limit their impact, these results demonstrate that other sources could be used to generate the high number of CAR iNKT cells that would be needed for clinical applications. However, further investigation into their efficacy in clinical trials is still needed, as while clinical trials using autologous CAR iNKT cells have been completed previously, evidence for the clinical safety and efficacy of allogeneic CAR iNKT cells remains limited.

A clinical trial using autologous CAR iNKT by Heczey et al. saw at least partial response in three out of twelve patients, with one of these patients escalating to complete response after a second dose was administered [123, 124]. Clinical applications of allogeneic CAR iNKT cells have only recently begun with several early phase clinical trials currently in progress [35, 125, 126]. A clinical trial in progress (ANCHOR, NCT00840853) using allogeneic CAR iNKT cells targeted to CD19 for treatment of B cell malignancies has shown highly promising preliminary results, with all patients tolerating the treatment well, and six out of twelve total patients achieving at least a partial response [126]. Further clinical research in this field is ongoing, and much more evidence will be needed before it can be concluded if allogeneic CAR iNKT can provide an effective off-the-shelf immunotherapeutic. While these early clinical trial results are encouraging, they also underscore a limitation shared across all ex vivo manufactured CAR cell platforms – regardless of the cell type used, the manufacturing process itself remains a bottleneck.

Even when effector-cell choice reduces alloreactivity (NK cell-based and iNKT cell-based platforms) or when gene engineering reduces alloreactivity (allogeneic T cell-based platform), most current products still rely on ex vivo genetic modification, expansion, and centralized manufacturing, which contributes to cost and logistical constraints. These shared limitations drive interest in approaches that minimize reliance on ex vivo manufacturing, such as in vivo CAR generation and delivery. In vivo CAR generation involves directly engineering immune cells within the patient, a topic that will be explored in the subsequent section.

## In vivo CAR generation and delivery

**Fig. 4:**
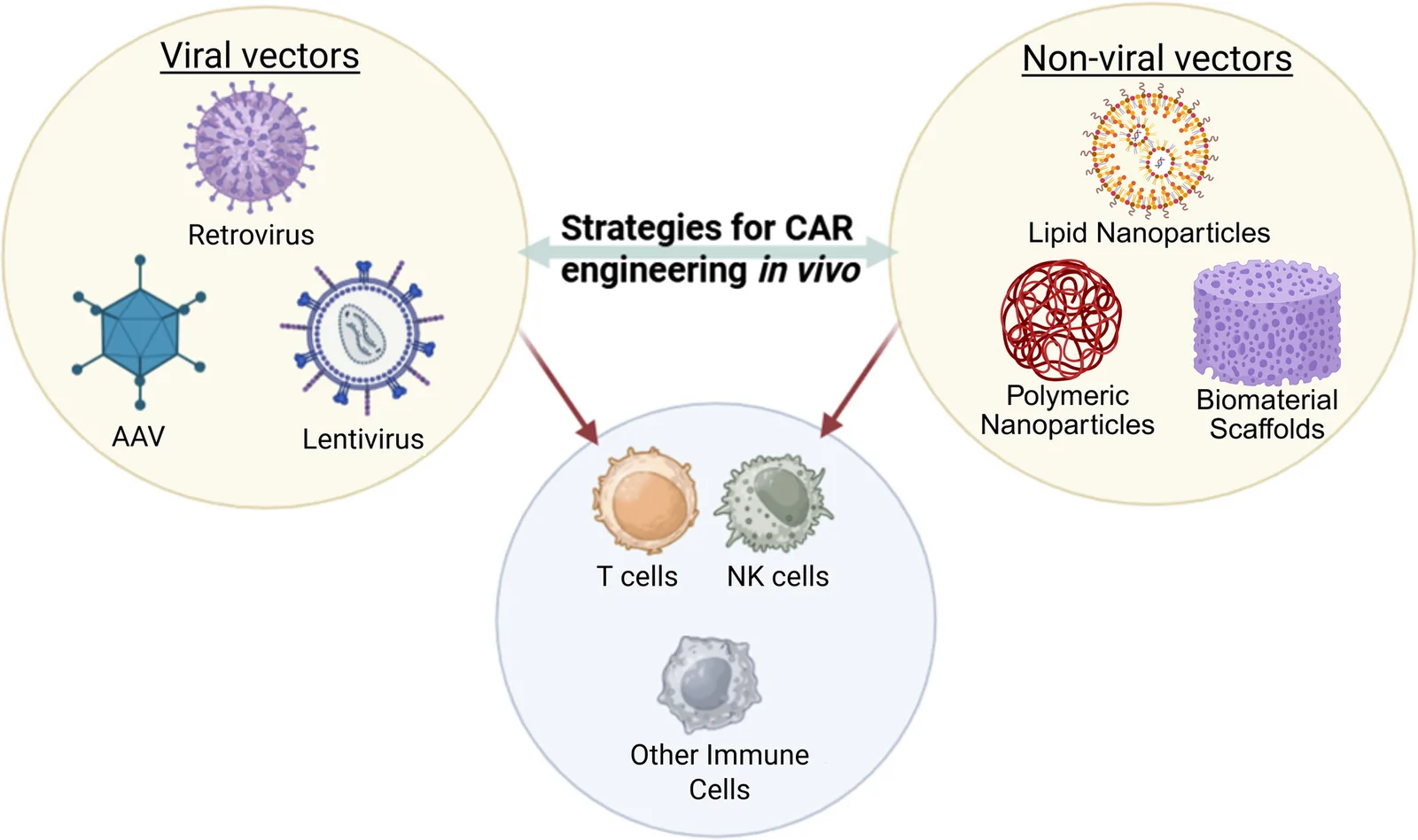
Schematic diagram of strategies for generating CAR constructs in vivo. These are classified into two main categories: viral vectors and non-viral vectors. These approaches can be used for T cells, NK cells, and other immune cell populations. Created in BioRender. Barlow, B. (2026) https://BioRender.com/j71n086

In vivo CAR engineering is emerging as a complementary off-the-shelf strategy that reframes adoptive cell therapy as a delivery and control problem, rather than ex vivo cell manufacturing problem. Instead of producing a patient-specific engineered cell product, in vivo approaches aim to deliver genetic payloads that program immune cells directly within the patient, potentially reducing time to treatment, simplifying supply chains, and lowering costs [127]. Conceptually, this approach standardizes the engineering reagent (vector or nanoparticle), rather than engineered cells themselves, while shifting key constraints toward cell-type specificity, expression control, and systemic safety [127]. These in vivo CAR engineering strategies are being explored across multiple immune cell types, including T and NK cells and, in some cases, other immune populations [127–129]. In this section, we focus primarily on T and NK cell programming as they most directly parallel ex vivo CAR platforms discussed above. Across modalities, in vivo CAR generation must overcome several shared technical challenges: achieving efficient intracellular delivery, restricting gene transfer to the desired immune cell subset, and controlling CAR expression kinetics to balance efficacy with toxicity risk [130]. Broadly, current systems can be grouped into viral transduction and non-viral transfection platforms, each offering distinct tradeoffs between transduction efficiency, manufacturability, immunogenicity, and control over payload persistence [130] (Fig. 4).

### Viral transduction

Viral vectors such as γ-retrovirus, lentiviral vectors (LVs), and adeno-associated viruses (AAVs) are widely used for ex vivo CAR manufacturing because of their high transduction efficiency and robust CAR expression [130]. Due to their reliability in ex vivo CAR generation, these vectors have also been explored for the introduction of CAR constructs in vivo, where the central challenge becomes maintaining efficiency while improving targeting precision and safety.

Preclinical studies support the feasibility of viral vector CAR delivery but also highlight control and safety constraints. In a study by Pfeiffer et al., LVs were used to produce CD19-targeting CD8^+^ CAR T cells in vivo in mice xenografted with Raji lymphoma [131]. At 7 days post LV injection, the frequency of CD8^+^ CAR T cells was 30%–50% in peritoneal fluid, 5%–30% in blood, and 10%–35% in spleen with elimination of lymphoma cells in the xenograft mice model [131]. However, this approach also induced cytokine elevations consistent with CRS in the model, underscoring that potent in vivo CAR generation can reproduce the same on-target immune toxicities observed with ex vivo products if CAR activity is not tightly controlled [131].

In another study, Andorko et al. presented INT2104, an engineered lentiviral platform designed for targeted in vivo delivery of genetic material to generate CAR T and CAR NK cells without lymphodepletion [132]. The system relies on a modified vesicular stomatitis virus G protein detargeted from its native low-density lipoprotein receptor and combined with a CD7-specific binding molecule. The CD7‑specific binder then redirects the vector to CD7⁺ T and NK cells, enabling selective transduction. In vitro, INT2104‑transduced PBMCs produced functional CAR20⁺ effector cells capable of antigen‑dependent killing of CD20⁺ B‑cell lines and robust cytokine secretion. In humanized mice, a single IV dose generated CAR⁺ T and NK cells and induced rapid, complete B‑cell depletion [132]. In another LV-based in vivo CAR study, anti-CD19 CAR T cells were generated using CD8-targeted LVs in a CD19^+^ Nalm-6 leukemia humanized mouse model, completely eliminating the tumor cells, and the CAR expression was shown in additional lymphocyte populations beyond T cells, including NK cells (i.e., CAR NK generation) [133]. While such “multi-lineage” programming may contribute to antitumor activity, it also illustrates a key limitation of viral in vivo delivery: off-target transduction can complicate safety, introduce variability in which effector cells are generated, and make dosing and toxicity harder to predict [134–137].

These observations emphasize the need for delivery platforms that retain high transduction efficiency while improving safety and targeting precision. One potential alternative are AAVs, as AAV vectors are primarily non-integrating and typically persist episomally, with durability that is context-dependent [130, 138]. AAVs have extensive prior use in gene therapy [139, 140], supporting their translational attractiveness, but in vivo CAR applications remain relatively limited to date. Nawaz et al. used AAVs to successfully produce anti-CD4 CAR T cells in a mouse model of human T cell leukemia, resulting in tumor regression [141]. Another study reported an in vivo, site-specific engineering of human T cells by combining anti-CD3-targeted enveloped delivery vehicles carrying Cas9-ribonucleoprotein with an AAV-mediated homology-directed repair [142]. This system enabled precise TRAC locus knock-in of a CAR transgene directly inside the subject, generating physiologically regulated CAR T cells without ex vivo manipulation. In humanized mouse models, a single injection produced robust CAR T‑cell expansion, sustained B‑cell depletion, and potent antitumor activity against B-cell acute lymphoblastic leukemia [142].

Nevertheless, a major remaining need across AAV-based approaches is rigorous characterization of cell-type specificity and off-target expression, as well as strategies to overcome pre-existing anti-AAV immunity that can reduce transduction efficiency and durability [141, 143]. More broadly, while viral delivery offers high efficiency and potentially durable expression, it faces constraints in large-scale GMP production, cost, and immunogenicity, motivating parallel development of non-viral platforms that can simplify manufacturing and improve control [130, 144].

### Non-viral vectors

Non-viral systems aim to deliver CAR payloads without viral components, potentially simplifying manufacturing, lowering cost, and reducing risks associated with viral production [130, 143]. In this context, the key differentiator across approaches is the delivery vehicle: in other words, how the system navigates biodistribution, engages the intended immune cell subset, enters cells, and releases CAR DNA or mRNA intracellularly [143]. Most reported non-viral platforms for in vivo CAR engineering fall into three vehicle families: lipid nanoparticles (LNPs), polymeric nanoparticles (PNPs), and biomaterial scaffold/depots.

LNPs represent a widely used nanocarrier architecture for nucleic acid delivery and are often treated as the reference platform due to their manufacturability and established formulation toolbox, highlighted by their use in COVID-19 mRNA vaccines [145]. One example by Chen et al. demonstrates that LNPs carrying CD19 CAR mRNA can target T cells, reprogramming them to eliminate leukemic cells from the bloodstream [146]. Another study by Zhou et al. demonstrated that delivering not only CD19 CAR mRNA but also shRNA targeting IL-6 using LNPs resulted in a more effective immunotherapeutic response compared to CD19 CAR T cells generated ex vivo [147].

A study by Rurik et al. demonstrated a fully in vivo approach to generating transient CAR T cells using LNPs to treat cardiac fibrosis, a major driver of heart failure [148]. The authors engineered CD5‑targeted LNPs to deliver modified mRNA encoding a fibroblast‑activation‑protein CAR directly into circulating T cells. After IV administration, T cells rapidly expressed the CAR, producing a short‑lived but therapeutically active CAR T population without ex vivo manipulation or permanent genomic modification. In mouse models of cardiac injury, these in vivo–generated CAR T cells selectively eliminated activated cardiac fibroblasts, leading to marked reductions in myocardial fibrosis and restoration of cardiac function, including improved ventricular remodeling and systolic performance [148]. This strategy could likely be modified for other CAR T cell therapies as well, such as for cancer immunotherapy, by using mRNA encoding for the desired CAR construct instead.

Hunter et al. used a similar strategy to create a fully in vivo CAR T engineering platform using targeted lipid nanoparticles (tLNPs) to deliver CAR‑encoding mRNA directly to T cells [149]. The nanoparticles were functionalized to target specific CD8⁺ T cell subsets, again enabling selective reprogramming without ex vivo manipulation or lymphodepleting chemotherapy. In human samples, tLNPs efficiently reprogrammed CD8⁺ T cells from both healthy donors and autoimmune patients, generating CAR T cells capable of killing autologous B cells. In humanized mouse models, in vivo dosing produced robust tumor control, and in cynomolgus monkeys, tLNP administration induced rapid and near‑complete B‑cell depletion, followed by reconstitution with predominantly naïve B cells, suggesting an immune “reset” [149]. These studies demonstrate that LNP platforms could allow for in vivo CAR T cell generation without the logistical and safety burdens created by ex vivo manufacturing. However, the performance of LNPs is tightly coupled to in vivo biodistribution and off-target uptake, which can drive dose constraints and complicate translation [138].

Furthermore, inherently short-lived expression of LNP-based in vivo CAR delivery necessitates higher or repeated systemic dosing, thereby increasing the risk of dose-limiting adverse effects [138]. In preclinical and clinical settings, systemically administered LNPs have been associated with transient innate immune activation and, in some cases, elevations of liver enzymes consistent with hepatotoxicity [150]. Moreover, effective mRNA doses can vary substantially across disease contexts, delivery routes, and animal models, complicating dose optimization and expanding the experimental space required to define therapeutic windows and safety margins [138, 151].

**Fig. 5:**
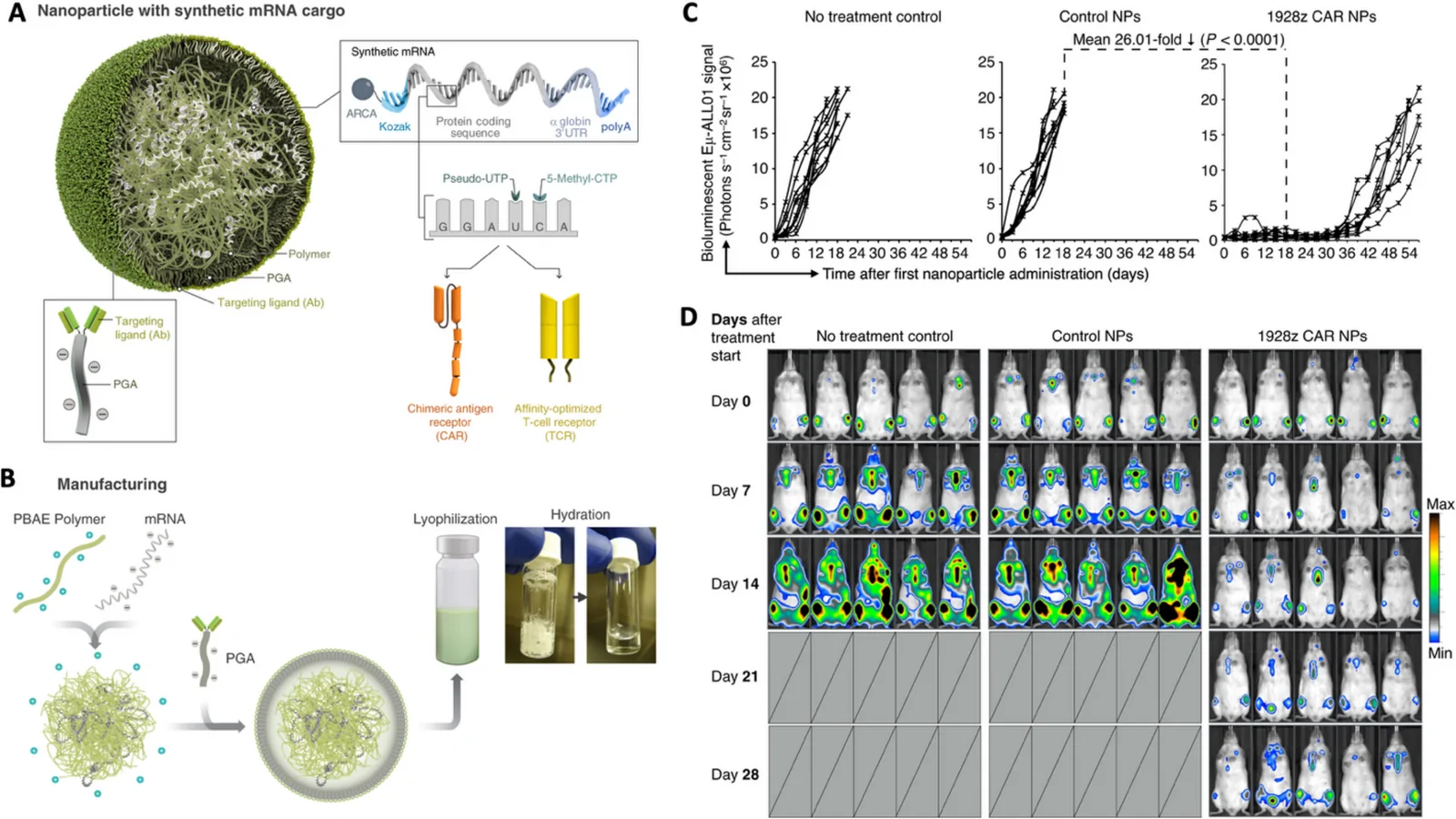
Polymer nanoparticle (PNP)-based in vivo CAR delivery to T cells. **A** Schematic of PNPs functionalized with anti-CD8 for T cell binding/uptake, a PGA anionic shield to reduce off-target binding, a biodegradable poly-(β-amino ester) matrix to protect and release mRNA, and encapsulated CAR mRNA for transient CAR expression. Once the PNPs were infused into the patient’s circulation, the PNPs transfer the transgenes they carry into the lymphocytes and transiently reprogramed the cells to express the disease-specific CARs on their surfaces. **B** Diagram describing how the nanoparticles were fabricated. **C** Plots of luciferase signal intensities after CAR-PNP injections along with no-treatment control and PNPs without CAR mRNA. Each line represents one animal, and each dot reflects its whole animal photon count. **D** Sequential bioimaging of firefly luciferase-expressing leukemia cells systemically injected into albino C57BL/6 mice. Reproduced with permission [151]. Copyright 2020, Springer Nature

PNPs offer a highly tunable alternative vehicle class, where polymer chemistry can be adjusted to influence stability in circulation, uptake by immune cells, and intracellular release [152, 153]. Several examples illustrate how polymer vehicles can be adapted for in vivo CAR delivery through formulation and targeting design. For example, Parayath et al. demonstrated the use of poly-(β-amino ester) polymer to fabricate PNPs that are capable of delivering CAR mRNA [151]. In mouse models of human leukemia, hepatitis B-induced hepatocellular carcinoma, and prostate cancer, repeated administration of poly-(β-amino ester) PNPs loaded with each targeted CAR mRNA induced enough CAR T cells in the host to result in disease regression (Fig. 5). In a separate example emphasizing persistent reprogramming via a PNP, Smith et al. designed CD3-targeted PNPs carrying DNA plasmids encoding CAR genes along with a hyperactive transposase enzyme called the piggyBac transposase, enabling in vivo generation of CAR-expressing T cells in a leukemia mouse model [154]. It is worth noting that this delivery system with transposase enzymes allows for persistent CAR expression, unlike single CAR mRNA approaches, without the need for viruses, thereby inducing in vivo reprogramming with less concerns of multiple dose injection.

Another emerging and interesting approach moves away from fully systemic dosing toward localized, bioinstructive delivery depots [127]. Here, biomaterial scaffolds act as a physical carrier that concentrates vectors and cells in a defined microenvironment, supporting immune-cell recruitment, activation, and subsequent release of engineered effectors [130, 155]. In one study by Agarwalla et al., an implantable Multifunctional Alginate Scaffold for T cell Engineering and Release (MASTER) was demonstrated [156]. The scaffold in MASTER functions as the delivery vehicle and provides a supportive niche via immobilized activation cues (anti-CD3/anti-CD28), enabling local cell expansion and release of functional CAR T cells in a xenograft lymphoma mouse model [156]. In this study, the scaffold was loaded with a retroviral vector; however, the delivery concept is directly applicable to non-viral in vivo CAR delivery. The scaffold functions as a modular, localized carrier and could, in principle, be loaded with non-viral vectors. Thus, it is best viewed as a platform for localized in vivo programming, independent of whether the genetic cargo is delivered via viral or non-viral modalities. Taken together, the delivery architectures discussed, from systemic LNPs and viral vectors to localized biomaterial scaffolds, reflect the breadth of strategies now advancing toward clinical translation for in vivo CAR cell generation.

### Clinical translation progress of In Vivo CAR delivery

Numerous clinical studies implementing in vivo CAR therapy strategies are currently registered and ongoing, with the majority of these trials targeted towards B-cell malignancies and autoimmune diseases [157]. However, limited evidence about their safety and efficacy in these trials is currently published. INT2104, a lentiviral platform for in vivo CAR delivery discussed earlier in this review, is currently undergoing a Phase I clinical trial for the treatment of patients with refractory/relapsing B-cell malignancies (NCT06539338) [157]. In another ongoing Phase I clinical study, Xu et al. reported an in vivo CAR T therapy, ESO‑T01, that was used in four adults with relapsed or refractory multiple myeloma [158]. ESO-T01 uses a T cell-targeted, immune shielded LV to deliver an anti-BCMA-CAR transgene directly into endogenous T cells after a single intravenous infusion and without lymphodepletion. Preliminary results from this trial stated that all four patients experienced acute inflammatory reactions after treatment, including fever, chills, hypotension, and transient hypoxia; three developed grade 3 CRS, and one developed grade 1 neurotoxicity, all resolving with corticosteroids and supportive care. Anti-BCMA CAR T cells were first detected on days 4 to 8 post-infusion and were then found to be further expanded across blood, marrow, and tumor sites, peaking in number from days 10 to 17. By days 28 to 60, two patients achieved complete responses and two achieved partial responses, with evidence of BCMA^+^ plasma‑cell clearance and robust intratumoral T‑cell infiltration [158].

Several trials are also in progress using non-viral platforms, predominantly LNPs [159]. While most of these trials are targeted at hematological malignancies, two in vivo CAR cell therapies developed by CREATE Medicines are aimed at treating solid tumors by introducing CAR constructs in myeloid cells using mRNA-containing LNPs (NCT05969041, NCT06478693) [157, 159, 160]. Preliminary results from one of these trials demonstrated that CAR expression was induced in targeted myeloid cells within hours post-infusion, and CAR^+^ myeloid cells were found localized into the TME [157, 160]. Despite the promising early results from these clinical studies, the field is still very new, and significantly more evidence is needed before the clinical translation of these technologies can be realized.

Nonetheless, the preclinical and clinical examples discussed suggest the possibility of in vivo CAR delivery for off-the-shelf cancer immunotherapy. In vivo CAR engineering has the potential to simplify manufacturing, reduce costs, and expand global access to CAR-based treatments. By eliminating the need for complex ex vivo isolation, activation, genetic modification, and reinfusion, in vivo approaches could transform CAR therapy into a more standardized and scalable platform. However, several critical challenges need to be addressed before clinical translation is possible. Both viral and non-viral vectors must achieve high specificity for desired immune cell targets as off-target gene transfer could compromise safety and introduce variability in therapeutic outcomes. Additionally, current methods involve either stable integration of CAR transgenes with risk of insertional mutagenesis and prolonged immune activation, or transient expression that requires repeated dosing to maintain therapeutic efficacy. Finding a way to balance between these systems will likely be a major focus of future research to enable tunable control of in vivo CAR delivery and expression.

## Summary and outlook

The rapid evolution of off-the-shelf cell-based immunotherapies reflects a shift from proof-of-concept efficacy trials to more scalable, standardized, and accessible solutions. As discussed throughout this review, allogeneic CAR T cells, engineered-NK cells, allogeneic CAR iNKT cells, and in vivo CAR generation strategies each address key limitations with current autologous therapies, but none has yet fully resolved the challenges of durability, safety, manufacturability, and cost. The next phase of innovation will likely involve a combination of these platforms to create optimal therapeutics. Ex vivo engineered allogeneic cells offer precise genome control, extensive quality testing, and well-defined product characterization prior to infusion, which are all attributes particularly advantageous for durable persistence, controlled phenotype selection, or multiplex gene edits that reduce alloreactivity and enhance tumor targeting. Allogeneic CAR T cells, NK cells, and iNKT cells have demonstrated encouraging preliminary safety and efficacy in early clinical trials, supporting the feasibility of off-the-shelf cell-based immunotherapies produced ex vivo. At the same time, in vivo CAR generation offers logistical simplicity, reducing manufacturing complexity, and potentially lowering costs by transforming the patient into the site of cell engineering. Transient mRNA-based delivery systems, polymeric nanoparticles, and scaffold-based approaches introduce tunable layers of control, enabling temporal restriction of CAR activity where permanent genome modification may not be desirable. For certain applications where prolonged immune activation carries risk, transient CAR expression may in fact represent a safety advantage rather than a limitation.

Nevertheless, it is important to acknowledge the major unresolved challenges facing off-the-shelf cell therapy platforms. Despite substantial bioengineering efforts to enable allogeneic T cell transfer, risks of GvHD and HvG rejection remain, particularly in settings that require repeated dosing. These constraints motivate increasingly complex genetic and synthetic modifications, which can inevitably reduce cellular fitness, increase product heterogeneity, and raise safety and regulatory burdens. NK cell-based therapeutics, while less constrained by GvHD, continue to face limitations in in vivo expansion and persistence, inefficient tumor trafficking, and vulnerability to immunosuppressive signals within the TME. Allogeneic CAR iNKT cells present a promising intermediary approach, leveraging the low GvHD risk provided by the invariant TCR without the need for HLA matching or additional genetic modification, yet this platform similarly deals with challenges in large-scale manufacturing and limited persistence following adoptive transfer. Across all three lineages, a central tradeoff is emerging between potency and controllability, in other words, strategies that maximize persistence and activation may enhance efficacy but can also increase the risk of systemic toxicities, motivating designs that tune durability and activation thresholds to the clinical context.

Because off-the-shelf cell therapies are evaluated as standardized biologics, their clinical translation will be shaped by manufacturability and product comparability at least as much as by receptor design. Enabling requirements include closed and scalable production workflows, robust cryopreservation with reliable post-thaw recovery, and stringent lot-to-lot consistency across donor-derived inputs or master cell banks. A continuing limitation is the lack of broadly accepted potency assays that reliably predict clinical activity, particularly for NK cell products, where cytotoxicity, serial killing capacity, metabolic fitness, and resistance to exhaustion may not correlate with conventional surface phenotyping. For engineered and gene-edited platforms, regulatory assessment will increasingly emphasize genomic integrity, stability of the engineered phenotype after expansion, and mitigation strategies for unintended edits or aberrant growth. For in vivo programming approaches that function as off-the-shelf engineering reagents, analogous translational requirements include rigorous characterization of biodistribution, cell-type specificity, and expression kinetics, supported by a safety case that addresses off-target transfection/transduction and innate immune activation.

Overall, off-the-shelf cell-based cancer immunotherapy is shifting from proof-of-concept engineering toward platform optimization under practical constraints, including predictable dosing, scalable manufacturing, and precise control of cellular behavior in vivo. Allogeneic T cell products may ultimately be most competitive in indications requiring high per-cell potency, provided alloreactivity and host immune rejection can be mitigated without compromising cellular fitness. NK cell-based therapies may achieve broader early adoption in settings that prioritize safety and enable repeat dosing; however, improvements in in vivo persistence, trafficking, and tumor microenvironment conditioning will likely be required for durable activity, particularly in solid tumors. iNKT cell-based strategies offer an innately allogeneic-compatible alternative by virtue of their MHC-independent tumor recognition, though achieving consistent in vivo expansion and persistence will likely be necessary to fully realize their therapeutic potential. In parallel, in vivo programming strategies extend the off-the-shelf paradigm by standardizing the engineering reagent rather than the engineered cell product, with clinical feasibility contingent on cell-type specificity and tunable expression kinetics. In the future, with ongoing preclinical and clinical efforts, platforms that enable precise control over biodistribution, activation thresholds, and persistence are well positioned to deliver more predictable efficacy with improved safety, supporting broader clinical adoption.

## Data Availability

No new datasets were generated during this study and all data supporting the conclusions are available in the cited literature.
